# Axial length changes and baseline predictors over five years in children treated with orthokeratology lenses: a retrospective cohort study

**DOI:** 10.3389/fbioe.2026.1870246

**Published:** 2026-08-12

**Authors:** Zixun Wang, Kaihua Xu, Xiaoling Zhang, Weiping Lin, Bei Du, Ruihua Wei

**Affiliations:** 1 Tianjin Key Laboratory of Retinal Functions and Diseases, Tianjin Branch of National Clinical Research Center for Ocular Disease, Eye Institute and School of Optometry, Tianjin Medical University Eye Hospital, Tianjin, China; 2 Handan Eye Hospital (The Third Hospital of Handan), Handan, Hebei, China

**Keywords:** axial length, baseline age, five-year follow-up, myopia progression, orthokeratology, predictive factors

## Abstract

**Background:**

Orthokeratology lenses (Ortho-K) are widely used in pediatric myopia management. However, axial length (AL) changes over 5 years, and the baseline predictors in treated cohorts remain insufficiently characterized.

**Methods:**

This retrospective cohort study included 656 right eyes of children who were treated with Ortho-K lenses and were followed for 5 years. Baseline variables included sex, age, refractive parameters (Cycloplegic Spherical Equivalent (SE), cycloplegic spherical power (DS), cycloplegic cylindrical power (DC)), keratometric indices, and AL. All refractive measurements were recorded in minus-cylinder notation. AL was assessed at baseline and at follow-up visits at 1, 3, and 5 years. Linear regression was used to analyze continuous 5-year changes in AL. Logistic regression was additionally performed using a prespecified axial elongation criterion (≤0.2 mm/year; ≤1.0 mm over 5 years) for within-cohort stratification.

**Results:**

Over 5 years, the mean AL elongation was 0.75 ± 0.49 mm in this cohort of children treated with Ortho-K lenses. In univariable analyses, baseline age, sex, SE, DS, and baseline AL were associated with the five-year change in AL. In the multivariable models, younger age, sex, and refractive status remained associated with five-year AL change, whereas baseline AL was not independently associated after adjustment. In the stratified logistic analyses, DS was associated mainly with the 1-year outcome grouping, whereas baseline age remained the most consistent factor over longer follow-up.

**Conclusion:**

This retrospective cohort study describes AL changes over 5 years and their baseline predictors in myopic children treated with Ortho-K lenses. Among the baseline variables, younger age was the most consistent factor associated with greater AL elongation during follow up, while sex and baseline refractive status showed less consistent associations. Due to the absence of an untreated or alternative treatment control group, this study cannot directly quantify the treatment effect of Ortho-K relative to natural myopia progression. These findings should therefore be interpreted as within cohort observational data and support closer AL monitoring and individualized management for younger children receiving Ortho-K treatment.

## Introduction

Myopia is currently a major global public health issue (Chen et al., 2023; Burton et al., 2021; Holden et al., 2016). A recent study in China indicates that under current epidemiological trends, the overall prevalence rates of myopia and high myopia will reach 61.3% and 17.6%, respectively, by 2050 (Pan et al., 2025). The elongation of the axial length (AL), as a key parameter in the progression of myopia, is considered a major cause of changes in retinal structure and even irreversible damage to visual function (Tao et al., 2025; Liu et al., 2025; Hayashi et al., 2010). Monitoring changes in AL has become one of the key indicators for assessing the effectiveness of myopia control. More clinical evidence is needed to guide the progression control of myopia in children.

In recent years, alongside advocacy of lifestyle changes, such as outdoor activities for children (Chen et al., 2025; Li et al., 2025), the range of myopia prevention and control methods has gradually expanded. These include repeated low-intensity red light therapy, defocus glasses, low-concentration atropine eye drops, multifocal soft contact lenses, and orthokeratology lenses (Ortho-K lenses) (Xiong et al., 2024; Liu et al., 2024; Zhang et al., 2024; Hazra et al., 2025; Queirós et al., 2025). Currently, there is a global consensus that Ortho-K lenses demonstrate effective control of AL progression in myopia. Some studies have also conducted follow-up observations on AL progression after Ortho-K lenses treatment. Our team’s previous study conducted an efficacy analysis on children 3 years after Ortho-K lenses treatment (Hu et al., 2025). The long-term therapeutic effects of Ortho-K lenses have yet to be substantiated by relevant studies regarding their sustained efficacy in controlling myopia (Martínez-Pérez et al., 2025).

The baseline ocular characteristics of children wearing Ortho-K lenses are often considered to influence treatment outcomes (Xia et al., 2025). Baseline factors such as age, sex, and height (Zhong et al., 2025), along with refractive parameters such as Cycloplegic Spherical Equivalent (SE), cycloplegic spherical power (DS), and AL, have been reported to influence the therapeutic outcomes of Ortho-K lenses (Chong et al., 2025; Han et al., 2025). However, as mentioned earlier, long-term Ortho-K lenses lack sufficient evidence to substantiate their efficacy, including studies examining these influencing factors.

Therefore, the purpose of this study was to describe AL changes over 5 years in children treated with Ortho-K lenses and to investigate baseline factors associated with longitudinal AL elongation in this treated cohort. While several studies have reported on the 5-year efficacy of Ortho-K lenses, many were characterized by relatively small sample sizes or focused on diverse ethnic groups. Our study provides longitudinal data from a cohort of 656 eyes and offers a detailed analysis of baseline predictors of AL changes over 5 years in Chinese children treated with Ortho-K lenses.

## Methods

### Study ethics and data collection

The study was approved by the Ethics Committee of Tianjin Medical University Eye Hospital (2024KY-67) and conducted in accordance with the principles outlined in the Declaration of Helsinki. Informed consent was obtained from the children and their guardians for all procedures involving ophthalmic assessments and the collection of basic information.

We conducted a retrospective cohort study by identifying patients who initiated Ortho-K treatment between January 2020 and December 2020 to ensure a consistent five-year follow-up period by September 2025. All children were fitted with Ortho-K (Emerald Lenses; Euclid Systems Corp., euclidsys.com), which are manufactured using BOSTON EQUALENSES II (Oprifocona) material with an oxygen permeability (Dk) of 127 × 10^−11^ (cm^2^/s) (mL O_2_/mL × mmHg). The Ortho-K used in this study was a spherical four-zone lenses. Each segment’s lenses dimensions and curvature are individualized according to the patient’s parameters. Treatment adherence was defined as wearing the lenses for at least 8 h per night, at least six nights per week, as verified by biweekly parent-reported logs. The patient selection process is shown in Supplementary Figure S1, including the number of children initially screened, reasons for exclusion, and the final analytic cohort. To avoid inter-eye correlation bias, only the right eye of each included child was used for the primary statistical analysis (Hu et al., 2025). For baseline information on children, we included the following indicators: (1) Patient basic information: sex, baseline age; (2) Refractive information: All refractive measurements were recorded in minus-cylinder notation. Cycloplegic SE, cycloplegic spherical power (DS), cycloplegic cylindrical power (DC), AL, keratometry parameters including Steep Eccentricity (E), Flat E, Flat Keratometry (K), and Steep K, and pupil distance (PD). All ocular parameters were measured three times under cycloplegia by two experienced optometrists to obtain final values. Cycloplegia was induced with three drops of 1% cyclopentolate (or other specific drug used) administered 5 min apart. Refraction was performed at least 30 min after the final drop when the pupillary light reflex disappeared (Hu et al., 2025). Cycloplegic autorefraction was obtained using an autorefractor (KR-800; Topcon, Japan), and then subjective optometry was performed without cycloplegia on the following day to get the final refraction and to calculate the SE (SE was calculated as spherical plus 1/2 columnar). AL was measured without cycloplegia using a non-contact optical biometer (Lenstar LS900; Haag Streit AG, Haag Streit.com). Follow-up AL measurements were collected at 1, 3, and 5 years after treatment initiation. By calculating the differences between the three-year and five-year data points and the baseline values, we determined the AL changes over 3 years and 5 years. The AL examination procedure was as described above. In this study, a prespecified axial elongation criterion of ≤0.2 mm/year was used to stratify outcomes within cohorts. This threshold was applied as a pragmatic, consensus-informed clinical categorization rather than as an age-adjusted surrogate for untreated natural history (Du et al., 2025; Lanca et al., 2021; Li et al., 2023; Bullimore et al., 2025; CJEO Journal Chinese Journal of Experimental Ophthalmology, 2024). Therefore, this study defines participants meeting the prespecified axial elongation criterion as AL changes ≤ 0.2 mm after 1 year of Ortho-K treatment, ≤ 0.6 mm after 3 years, and ≤ 1.0 mm after 5 years, while participants not meeting the prespecified axial elongation criterion is defined as AL changes > 0.2 mm after 1 year, > 0.6 mm after 3 years, and > 1 mm after 5 years.

Exclusion criteria were as follows: (1) all subjects with missing baseline information or 5 years of AL data; (2) children with other ocular conditions such as strabismus, amblyopia, or ocular infections; (3) children who received additional myopia control interventions alongside Ortho-K treatment (such as defocus spectacles, low-concentration atropine eye drops, repeated low-level red-light therapy, and so on); (4) children who discontinued Ortho-K treatment or failed to adhere to prescribed wear protocols during treatment.

### Statistical analysis

Continuous variables were tested for normality using the Shapiro-Wilk (S-W) test. Data meeting normal distribution were presented as mean ± SD, while non-normal distributions were shown as median [P_25_, P_75_] (P_25_ stands for the 25th percentile, and P75 stands for the 75th percentile). Categorical variables were presented as counts and percentages. All statistical analyses were performed using GraphPad Prism 9.0 software (GraphPad Software, La Jolla, CA, USA). We conducted a simple linear correlation analysis to examine the linear relationship between baseline data and 5-year AL changes. Subsequently, we performed pairwise Pearson correlation analysis on significant continuous variables and evaluated the impact of significant variables on 5-year AL changes using multivariate linear regression. Variables that showed significant associations with 5 year AL change in univariable analyses, together with clinically relevant baseline factors, were considered for multivariable linear regression. Because SE and DS were highly correlated, they were entered into separate multivariable models. The SE based model included sex, age, SE, and baseline AL, and the DS based model included sex, age, DS, and baseline AL. Group comparisons were performed using Student’s t-test for normally distributed variables or the Wilcoxon rank-sum test for non-normally distributed data. Categorical variables were presented as counts and percentages, with group differences evaluated via the chi-square test or Fisher’s exact test, as appropriate. Logistic regression analyses were performed for 1-, 3-, and 5-year within-cohort outcome stratification based on the prespecified AL elongation criterion. P < 0.05 was considered significant.

## Results

### Descriptive statistics

The study included 656 right eyes (OD). The cohort comprised 44.82% males and 55.18% females. The median baseline age was 10.00 years (IQR: 9.00–11.00), and the median SE was −3.00 D (IQR: 4.25 to −2.00). Other baseline characteristics, such as corneal curvature (Flat K and Steep K), PD, and AL at baseline and follow-up time points, are detailed in Table 1.

**TABLE 1:** Baseline information included in this study.

Variables	Level	Overall
n	​	656
Sex (%)	Male	294 (44.82)
	Female	362 (55.18)
Age (median [IQR])	​	10.00 [9.00, 11.00]
SE (median [IQR])	​	−3.00 [-4.25, −2.00]
DS (median [IQR])	​	−2.75 [-3.75, −1.75]
DC (median [IQR])	​	−0.50 [-0.75, 0.00]
Flat e (median [IQR])	​	0.64 [0.57, 0.69]
Steep e (median [IQR])	​	0.47 [0.37, 0.57]
Flat K (mean (SD))	​	42.97 (1.19)
Steep K (mean (SD))	​	44.20 (1.39)
PD (median [IQR])	​	5.14 [4.65, 5.65]
Base AL (median [IQR])	​	24.70 [24.17, 25.33]
One year AL (median [IQR])	​	24.86 [24.40, 25.44]
Three years AL (median [IQR])	​	25.23 [24.70, 25.75]
Five years AL (median [IQR])	​	25.44 [24.91, 26.06]

SE: cycloplegic spherical equivalent; DS: cycloplegic spherical power; DC: cycloplegic cylindrical power; e: Eccentricity; K: keratometry; PD: pupil distance; AL: axial length.

### Regression analysis of AL changes over five years

**FIGURE 1:**
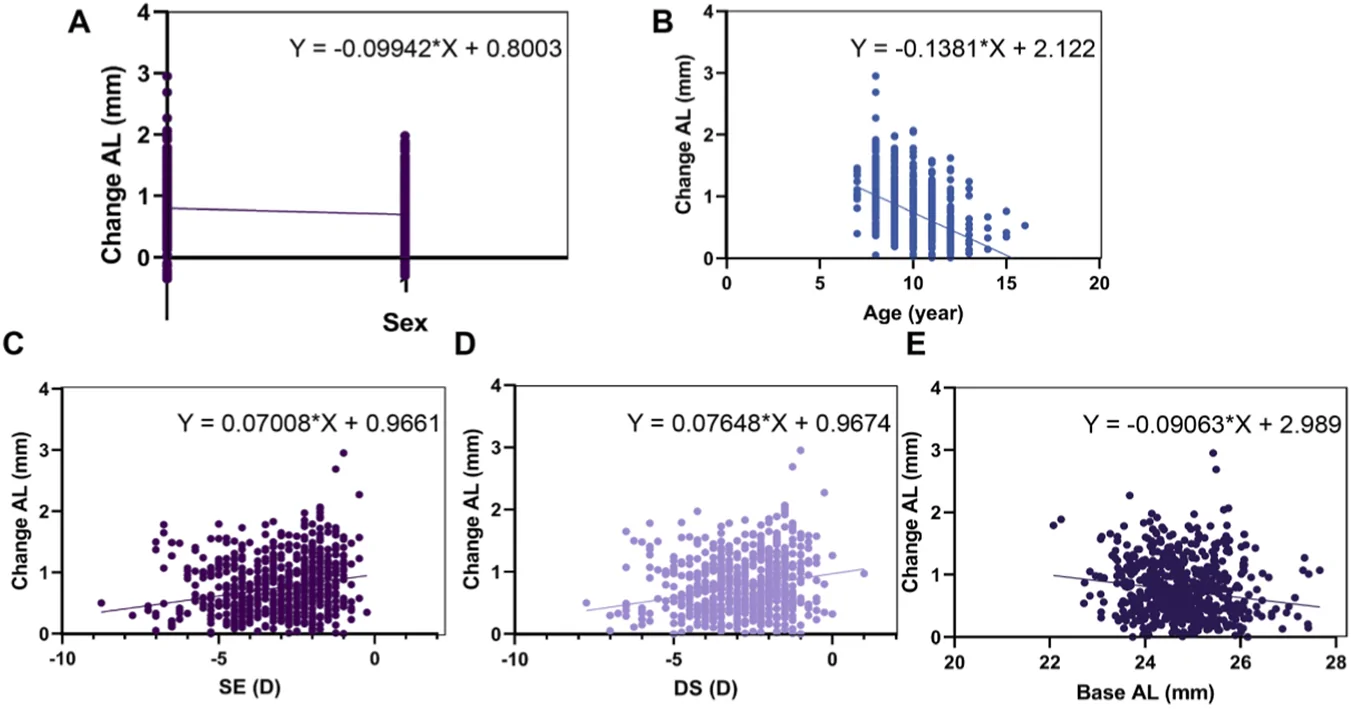
Linear relationship and equations between sex **(A)**, age **(B)**, SE (Cycloplegic Spherical Equivalent) **(C)**, DS: Cycloplegic spherical power; **(D)**, base AL (axial length) **(E)**, and change AL of 5 years.

**FIGURE 2:**
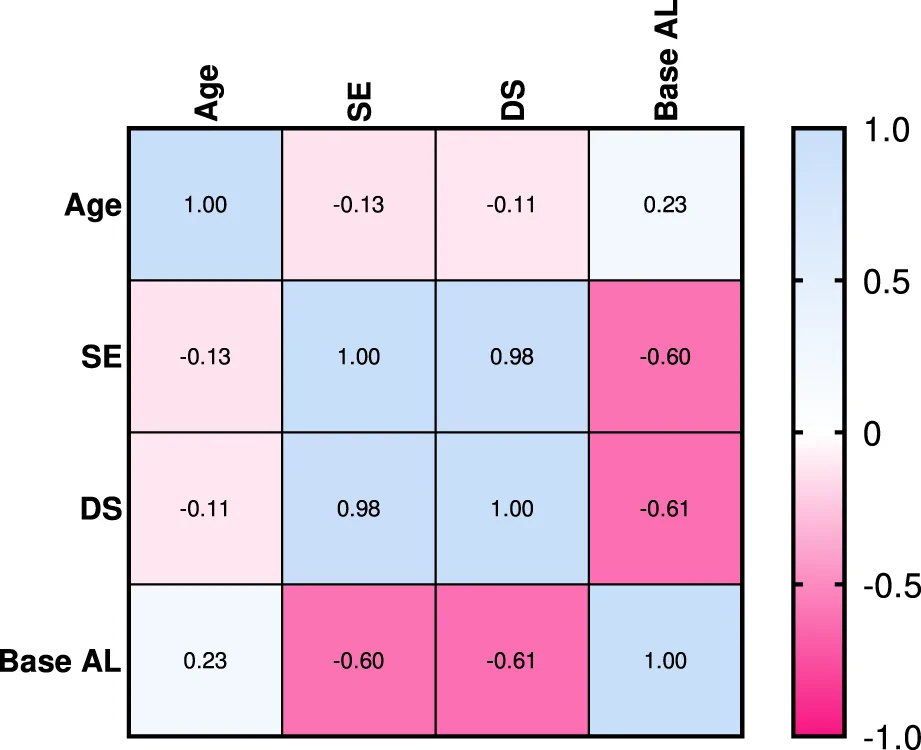
Correlation heatmap of meaningful parameter in univariate linear regression analysis. The heatmap illustrates pairwise Pearson correlation coefficients among age, SE (Cycloplegic Spherical Equivalent), DS (Cycloplegic spherical power), and Base AL (axial length). Blue indicates positive correlations, while pink indicates negative correlations, with color intensity reflecting the strength of the association (scale from −1 to +1).

Single-linear regression analysis (Table 2) identified several factors significantly associated with AL changes over 5 years: sex (*r* ^2^ = 0.0102, p = 0.01), baseline age (*r* ^2^ = 0.1993, p < 0.0001), SE (*r* ^2^ = 0.0454, p < 0.0001), DS (*r* ^2^ = 0.0485, p < 0.0001), and baseline axial length (base AL) (*r* ^2^ = 0.0249, p < 0.0001). In contrast, DC, Flat e, Steep e, Flat K, Steep K, and PD showed no significant associations (p > 0.05). Figure 1 illustrates the linear relationships between sex, baseline age, SE, DS, base AL, and five-year AL changes, supporting the regression findings. Supplementary Figure S2 further demonstrates that there are gender-specific differences in Change AL. Figure 2 displays a correlation heatmap highlighting positive and negative correlations between baseline age, SE, DS, and base AL, with base AL showing strong negative correlations with SE and DS. Since SE and DS are highly correlated (r = 0.98), we performed multi-linear regression analyses separately for Sex, Age, SE, and Base AL, and for Sex, Age, DS, and Base AL.

**TABLE 2:** Single-linear and Multi-linear Regression Analysis of baseline data versus AL changes over 5 years.

Variables	*r* ^2^	P (Single-linear)	β (SE)^*^	P (SE) (Multi-linear)	β (DS)^#^	P (DS) (Multi-linear)
Sex	0.0102	0.01	−0.1098	0.0022	−0.1013	0.0048
Age	0.1993	<0.0001	−0.1317	<0.0001	−0.1332	<0.0001
SE	0.0454	<0.0001	0.0594	<0.0001	​	​
DS	0.0485	<0.0001	​	​	0.0703	<0.0001
DC	0.0044	0.09	​	​	​	​
Flat e	0.0051	0.07	​	​	​	​
Steep e	0.0057	0.05	​	​	​	​
Flat K	0.0001	0.78	​	​	​	​
Steep K	0.0001	0.82	​	​	​	​
PD	0.0002	0.70	​	​	​	​
Base AL	0.0249	<0.0001	0.0088	0.74	0.0201	0.46

^*^The multivariate linear regression equation is: Y = 2.089 - 0.1098 * Sex - 0.1317 * Age + 0.05938 * SE + 0.008778 * Base AL (*R* ^2^ = 0.2374).

^#^The multivariate linear regression equation is: Y = 1.838 - 0.1013 * Sex −0.1332 * Age + 0.07034 * DS + 0.02005 * Base AL (*R* ^2^ = 0.2418).

Male: 0; Female: 1; SE: SE: cycloplegic spherical equivalent; DS: cycloplegic spherical power; DC: cycloplegic cylindrical power; e: Eccentricity; K: keratometry; PD: pupil distance; AL: axial length.

In the multivariate linear regression model (Table 2), two primary predictive equations were constructed: The SE-based model: Y = 2.089 - 0.1098 * Sex - 0.1317 * Age + 0.05938 * SE + 0.008778 * Base AL. The DS-based model: The multivariate linear regression equation is: Y = 1.838 - 0.1013 * Sex −0.1332 * Age + 0.07034 * DS + 0.02005 * Base AL. As shown in Supplementary Tables S1, 2, All VIF <2 indicates no significant multicollinearity among the independent variables. In the multivariable models, younger age, sex, and refractive status remained associated with five-year AL change, whereas baseline AL was not independently associated after adjustment. Specifically, younger age was associated with greater AL elongation, female sex with lower AL change, and lower SE or DS values with greater AL elongation. The *R* ^2^ values of the SE based and DS based models were 0.2374 and 0.2418, respectively, indicating modest explanatory performance for 5 year AL change.

**FIGURE 3:**
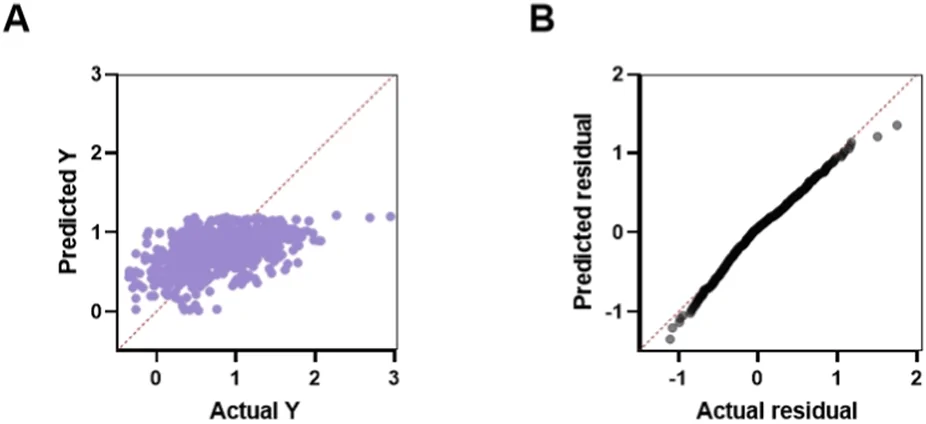
**(A)** Actual change AL of 5 years vs. predicted change AL plot: multi-linear regression (SE, Cycloplegic Spherical Equivalent). **(B)** QQ plot: Relationship between actual and predicted residuals base on multi-linear regression (SE).

**FIGURE 4:**
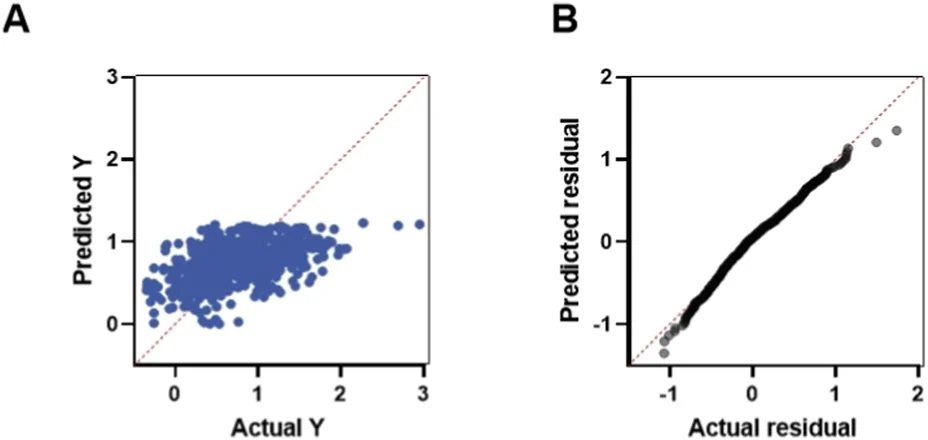
**(A)** Actual change AL of 5 years vs. predicted change AL plot: multi-linear regression (DS: cycloplegic spherical power). **(B)** QQ plot: Relationship between actual and predicted residuals base on multi-linear regression (DS).

Figures 3, 4 present scatter plots of predicted versus actual values and residual Q-Q plots for multivariate regression models based on SE and DS, respectively. The results demonstrate that the distributions of predicted and actual values are relatively concentrated, with residuals generally following a normal distribution, indicating that the models exhibit good predictive consistency and fit.

### Group-based regression analysis

Table 3 presents univariable and multivariable logistic regression analyses for 1-year, three-year, and five-year follow-ups. Over the 5-year follow-up period, the mean AL elongation was 0.75 ± 0.49 mm, corresponding to an average annual elongation of 0.15 mm/year. Using the prespecified AL elongation criterion (≤1.0 mm over 5 years) for within-cohort stratification, 464 of 656 children (70.7%) were classified as participants meeting the prespecified axial elongation criterion at the 5-year visit. The proportion of children meeting the prespecified axial elongation criterion decreased from 59.9% at 1 year to 62.3% at 3 years and 70.7% at 5 years. Baseline age was consistently significant in both univariable and multivariable analyses across all time points (p < 0.001), with lower baseline age associated with higher odds of AL change >0.2 mm/year. SE and DS were significant in univariable analysis at all time points (p < 0.01) but generally lost significance in multivariable analysis, except for DS at 1 year (OR = 1.89, p = 0.042). Base AL was significant in univariable analysis at one and 3 years (p < 0.001) but not in multivariable analysis or at 5 years. Other variables (sex, eye, DC, Flat e, Steep e, Flat K, Steep K, PD) did not show consistent significant associations. Because the prespecified ≤0.2 mm/year threshold was not age adjusted, the logistic regression findings should be interpreted as associations with threshold-based AL elongation status within this Ortho-K-treated cohort. This fixed criterion may classify younger children with naturally faster AL growth as having greater AL elongation, even when part of the observed growth reflects age-related ocular development.

**TABLE 3:** Single-factor and multi-factor regression analysis of the different groups.

Variables	​	Meeting the prespecified cumulative AL criterion	Not meeting the prespecified cumulative AL criterion	OR (univariable)	OR (multivariable)
One year		​			
Sex	Male	184 (46.8%)	110 (41.8%)	​	​
​	Female	209 (53.2%)	153 (58.2%)	1.22 (0.89-1.68, p = 0.208)	​
Age	Mean ± SD	10.3 ± 1.6	9.5 ± 1.4	0.72 (0.64-0.80, p <.001)	0.73 (0.65-0.82, p <.001)
SE	Mean ± SD	−3.5 ± 1.5	−2.6 ± 1.3	1.65 (1.45-1.87, p <.001)	0.85 (0.48-1.50, p = 0.570)
DS	Mean ± SD	−3.3 ± 1.4	−2.3 ± 1.2	1.72 (1.50-1.97, p <.001)	1.89 (1.02-3.50, p = 0.042)
DC	Mean ± SD	−0.2 ± 0.8	−0.5 ± 0.6	0.99 (0.94-1.04, p = 0.568)	​
Flat e	Mean ± SD	0.7 ± 0.2	0.6 ± 0.1	0.95 (0.79-1.14, p = 0.574)	​
Steep e	Mean ± SD	0.6 ± 0.2	0.5 ± 0.2	0.96 (0.82-1.12, p = 0.587)	​
Flat K	Mean ± SD	42.9 ± 1.2	43.1 ± 1.1	1.13 (0.99-1.29, p = 0.065)	​
Steep K	Mean ± SD	44.1 ± 1.5	44.3 ± 1.3	1.10 (0.98-1.23, p = 0.107)	​
PD	Mean ± SD	5.2 ± 0.7	5.3 ± 0.2	1.06 (0.94-1.20, p = 0.361)	​
Base AL	Mean ± SD	24.9 ± 0.9	24.5 ± 0.8	0.48 (0.39-0.60, p <.001)	0.80 (0.62-1.05, p = 0.108)
Three years					
Sex	Male	206 (50.4%)	122 (49.4%)	​	​
	Female	203 (49.6%)	125 (50.6%)	1.04 (0.76-1.43, p = 0.809)	​
Age	Mean ± SD	10.4 ± 1.6	9.3 ± 1.3	0.61 (0.54-0.69, p <.001)	0.60 (0.53-0.69, p <.001)
SE	Mean ± SD	−3.4 ± 1.5	−2.8 ± 1.4	1.31 (1.17-1.48, p <.001)	0.86 (0.50-1.50, p = 0.601)
DS	Mean ± SD	−3.1 ± 1.4	−2.6 ± 1.4	1.34 (1.19-1.51, p <.001)	1.61 (0.89-2.91, p = 0.115)
DC	Mean ± SD	−0.2 ± 0.8	−0.5 ± 0.6	0.98 (0.90-1.06, p = 0.617)	​
Flat e	Mean ± SD	0.7 ± 0.2	0.6 ± 0.1	0.95 (0.80-1.14, p = 0.596)	​
Steep e	Mean ± SD	0.6 ± 0.2	0.5 ± 0.2	0.96 (0.82-1.12, p = 0.610)	​
Flat K	Mean ± SD	43.0 ± 1.2	43.0 ± 1.2	1.04 (0.91-1.19, p = 0.573)	​
Steep K	Mean ± SD	44.2 ± 1.4	44.2 ± 1.4	1.01 (0.90-1.13, p = 0.861)	​
PD	Mean ± SD	5.1 ± 0.7	5.3 ± 0.3	1.06 (0.94-1.20, p = 0.337)	​
Base AL	Mean ± SD	24.9 ± 0.8	24.6 ± 0.9	0.71 (0.58-0.86, p <.001)	1.14 (0.87-1.48, p = 0.336)
Five years					
Sex	Male	234 (50.4%)	94 (49%)	​	​
	Female	230 (49.6%)	98 (51%)	1.06 (0.76-1.48, p = 0.731)	​
Age	Mean ± SD	10.3 ± 1.6	9.2 ± 1.3	0.61 (0.53-0.69, p <.001)	0.61 (0.53-0.70, p <.001)
SE	Mean ± SD	−3.3 ± 1.4	−2.9 ± 1.6	1.19 (1.05-1.34, p = 0.005)	0.85 (0.48-1.52, p = 0.589)
DS	Mean ± SD	−3.0 ± 1.4	−2.7 ± 1.5	1.20 (1.06-1.36, p = 0.004)	1.37 (0.75-2.52, p = 0.303)
DC	Mean ± SD	−0.2 ± 0.8	−0.6 ± 0.7	0.90 (0.68-1.20, p = 0.476)	​
Flat e	Mean ± SD	0.7 ± 0.2	0.7 ± 0.1	0.97 (0.83-1.13, p = 0.699)	​
Steep e	Mean ± SD	0.6 ± 0.2	0.5 ± 0.2	0.97 (0.83-1.13, p = 0.692)	​
Flat K	Mean ± SD	43.0 ± 1.2	43.0 ± 1.2	0.98 (0.85-1.13, p = 0.789)	​
Steep K	Mean ± SD	44.2 ± 1.4	44.2 ± 1.4	1.00 (0.88-1.12, p = 0.942)	​
PD	Mean ± SD	5.2 ± 0.7	5.3 ± 0.3	1.06 (0.95-1.18, p = 0.329)	​
Base AL	Mean ± SD	24.8 ± 0.8	24.7 ± 0.9	0.84 (0.69-1.03, p = 0.086)	​

SE: cycloplegic spherical equivalent; DS: cycloplegic spherical power; DC: cycloplegic cylindrical power; e: Eccentricity; K: keratometry; PD: pupil distance; AL: axial length.

The grouping criteria were based on cumulative AL, change from baseline: ≤0.2 mm at 1 year, ≤0.6 mm at 3 years, and ≤1.0 mm at 5 years. These thresholds correspond to a pragmatic criterion of ≤0.2 mm/year and were used for within cohort stratification.

## Discussion

This study describes longitudinal changes in AL and baseline predictors in a five-year cohort of Ortho-K-treated individuals. While the long-term effects of Ortho-K have been explored in several cohorts, our analysis of 656 eyes provides a detailed dataset to identify predictors of AL elongation in a large Chinese pediatric population. Among children treated with Ortho-K lenses, baseline age was a significant protective factor for inhibiting AL progression. Sex showed an independent association in the multivariate model, while baseline spherical power was significant in univariate analysis but lost significance after multivariate adjustment. In the short term (1 year), DS showed predictive value for AL growth, whereas baseline age exerted a dominant influence during long-term follow-up. These findings suggest that, among children treated with Ortho-K lenses, clinical management should incorporate stratified follow-up based on baseline age and individual ocular characteristics. Consistent with previous longitudinal reports, our findings align with long-term observations of length change in children treated with Ortho-K. While previous studies by Hiraoka et al. and Santodomingo-Rubido et al. have reported long-term outcomes, our study provides evidence from a substantially larger cohort, enabling a more robust analysis of baseline predictors in a Chinese population (Hiraoka et al., 2012; Santodomingo-Rubido et al., 2024). This expanded sample size enhances the statistical power to identify age and baseline refraction as clinically relevant baseline factors associated with longitudinal AL change.

This study describes AL changes at 1, 3, and 5 years in children treated with Ortho-K lenses. Previous long-term studies, including the report by Hiraoka et al., have described AL changes in children treated with Ortho-K lenses over a five-year period, and our findings are generally consistent with those longitudinal observations (Hiraoka et al., 2012). Furthermore, that study included only 29 children, whereas our dataset is considerably larger. However, a limitation is the absence of a control group with no myopia control intervention over such an extended 5-year period. In this study, an annual AL elongation of 0.2 mm was used as a pragmatic clinical cutoff for within-cohort stratification (He et al., 2023; Wang et al., 2024). A major limitation of our study is the absence of an untreated comparator group over the five-year follow-up period. Therefore, the observed AL changes should be interpreted as longitudinal findings within an Ortho-K treated cohort. They should not be regarded as definitive estimates of treatment effectiveness relative to untreated natural progression. Unlike our team’s previous research, we did not place excessive emphasis on AL regression when evaluating long-term AL control (Hu et al., 2025). Our earlier three-year study specifically highlighted the efficacy of AL regression. Considering that long-term cohort studies demonstrate that AL control outcomes have greater clinical relevance than regression rates, and that the mechanisms of AL regression appear to involve both the anterior segment and the retina, yet remain poorly understood, our current research prioritizes maintaining long-term AL control within safe parameters (Wang et al., 2022; Swiatczak et al., 2024; Chen and Shen, 2021; Hu et al., 2023).

Multi-linear regression analysis results indicate that changes in AL following 5 years of Ortho-K lenses treatment correlate with sex, baseline age, and SE or DS. Further classification of AL control outcomes at 1, 3, and 5 years of Ortho-K lens treatment, followed by single-factor and multi-factor regression analyses, revealed that the 1-year stratified outcome correlates with baseline DS and baseline age, whereas 3-year and 5-year stratified outcomes are independently associated with baseline age alone. These logistic regression results should be interpreted with caution because the prespecified ≤0.2 mm/year threshold was not age-adjusted. Axial growth is strongly age-dependent in children, and younger children may naturally exhibit faster AL elongation. Therefore, the fixed threshold may misclassify treatment response, particularly in younger children. The observed association between younger baseline age and the threshold-based outcome may partly reflect age-related ocular growth and individual differences in AL changes during Ortho-K treatment. Consistent with multiple systematic reviews and cohort studies, we found baseline age to be a core predictor of AL progression following Ortho-K lenses treatment, regardless of treatment duration (Martínez-Pérez et al., 2025; Han et al., 2025). T. M. Jakobsen et al. conducted a 3-year randomized controlled trial on the efficacy of Ortho-K lenses and found that no statistically significant difference in treatment efficacy was observed between children who initiated treatment at younger versus older ages, suggesting that age at treatment initiation may not diminish the overall control effect. This finding does not fully align with our research results (Jakobsen et al., 2025). A possible reason is that their study included only 19 participants, resulting in an insufficient baseline age distribution and sample size. Furthermore, other studies consistently indicate that older baseline age is an indicator of good control with Ortho-K lenses (Zhong et al., 2025; Santodomingo-Rubido et al., 2024). One possible explanation is that baseline age may reflect the stage of ocular growth. Younger children are more likely to be in a period of rapid physiological AL development, which may make them less likely to meet a fixed axial elongation criterion during follow-up, even under Ortho-K treatment (Grytz and El Hamdaoui, 2017; Yu and Zhou, 2022). Previous experimental and clinical studies suggest that age-related differences in ocular growth and scleral remodeling could be involved (Shirai et al., 2018). However, none of the relevant biomechanical properties (e.g., scleral collagen organization, collagen cross-linking, creep tendency, ocular rigidity, or other biomechanical properties) were directly measured in the present study (Queirós et al., 2025; Benavente-Perez, 2023). Therefore, the association between younger baseline age and greater axial elongation should be interpreted primarily as an observational association within this treated cohort. Any proposed biomechanical explanation remains purely hypothesis-generating and should not be regarded as an established mechanism. For younger children or those with lower baseline myopia, closer AL monitoring may be warranted, and combined treatment may be considered when clinically appropriate. However, the explanatory power of these multivariable models was modest. The SE based and DS based models explained approximately one quarter of the variability in 5 year AL elongation, suggesting that a substantial proportion of interindividual variability remained unexplained. Factors not included in the current models, such as objective adherence, lens centration, corneal reshaping response, near work, outdoor exposure, and other biological or environmental factors, may also contribute to long term AL changes. In addition, formal VIF based multicollinearity diagnostics were not performed, and confidence intervals for regression coefficients were not reported consistently. Although SE and DS were analyzed in separate models due to their high correlation, this should be taken into account when interpreting the regression results.

Sex also showed an association with five-year AL change in the multivariable linear model. Some previous studies have reported sex-related differences in AL progression or Ortho-K treatment outcomes, but the direction and magnitude of this association have not been consistent across populations, baseline refractive status, and follow-up durations (Chen et al., 2022; Fan et al., 2025). Potential explanations may include differences in ocular maturation, hormonal environment, visual behavior, or treatment adherence. However, estrogen levels, scleral biomechanical properties, near-work exposure, outdoor activity, and objective wear time were not assessed in this study (Gong et al., 2015). Therefore, sex-related biological explanations should be regarded as speculative, and the clinical relevance of this association requires further confirmation in prospective studies.

Baseline SE or DS was associated with five-year AL change in multivariable linear models, and DS was associated with the 1-year threshold-based outcome in logistic regression. The association between baseline SE or DS and AL changes in children treated with Ortho-K lenses may be partly related to the optical mechanism of peripheral myopic defocus. Ortho-K lenses reshape the central cornea and alter peripheral retinal defocus, and the magnitude of this optical change may vary according to baseline refractive status. This may explain why SE or DS showed associations with AL changes, whereas baseline age remained the most consistent factor over longer follow-up. However, peripheral refraction, lens centration, corneal reshaping pattern, and higher-order aberrations were not directly measured in this study; therefore, this mechanistic interpretation should be regarded as hypothesis-generating. In fact, longer baseline AL or higher initial myopia is often associated with slower subsequent progression, but this effect is frequently moderated by baseline age and other variables. Research indicates that Ortho-K lenses inhibit AL growth by altering the central corneal curvature and inducing myopic defocus in the periphery. Different behavioral or physiological factors, such as corneal response to reshaping, lenses centration, and changes in corneal higher-order aberrations, may influence the intensity of peripheral defocus. These findings are consistent with the possibility that baseline refractive status may be related to the optical or corneal reshaping response to Ortho-K treatment (Hu et al., 2023). However, because peripheral refraction, lens centration, corneal reshaping patterns, and higher-order aberrations were not directly quantified, this interpretation remains hypothesis-generating and should not be considered evidence of a specific mechanism.

This study has the following limitations: First, this was a retrospective cohort study of children treated with Ortho-K lenses, and an untreated control group was not included. Therefore, the study was designed to describe AL changes and associated baseline predictors within the treated cohort. Future multicenter prospective studies with appropriate comparator groups are needed to more precisely estimate the relative treatment effect. Selection bias and attrition bias should also be considered because the final analysis included only children with complete 5 year AL data and adequate Ortho-K adherence. Children who discontinued Ortho-K treatment, had poor adherence, received additional myopia control interventions, or lacked complete follow up data were excluded. These excluded children may have differed systematically from the included cohort, particularly if greater AL elongation, treatment failure, inconvenience, adverse events, or parental concern increased the likelihood of discontinuation or additional intervention. Therefore, the observed AL outcomes may be more favorable than those expected in real-world Ortho-K practice. Because adherence was assessed using parent reported logs rather than objective wear time monitoring, adherence misclassification cannot be excluded. This subjective assessment may have led to an overestimation of adherence and influenced the observed AL outcomes.

Second, whether the efficacy of Ortho-K lenses treatment correlates with choroidal parameters remains a viable area for future investigation. Future studies should incorporate additional retinal and choroidal parameters to comprehensively analyze their influence on long-term Ortho-K treatment outcomes. Third, all variables included in this study were measured at baseline. Future research could investigate whether continuous changes in AL can predict and indicate long-term AL control, potentially establishing an artificial intelligence predictive model for Ortho-K lenses treatment outcomes. Fourth, although we applied a prespecified ≤0.2 mm/year threshold for within-cohort AL elongation stratification, this criterion was not age-adjusted. Because AL growth is strongly age dependent in children, this fixed threshold may misclassify treatment response, particularly in younger children with naturally faster axial growth. Therefore, the logistic regression results based on this threshold should be interpreted cautiously. Future studies should incorporate age-specific AL growth references or age-adjusted criteria to provide a more precise assessment of longitudinal AL outcomes in children treated with Ortho-K lenses. Additionally, due to the relatively narrow baseline age range in this study, no subgroup analysis was conducted. Future studies will evaluate the long-term efficacy of Ortho-K lenses treatment in populations of different baseline ages and diopter strengths.

## Conclusion

This retrospective cohort study describes AL changes over 5 years and their baseline predictors in myopic children treated with Ortho-K lenses. Among the baseline variables, younger age was the most consistent factor associated with greater AL elongation during follow up. These findings support closer AL monitoring and individualized management for younger children receiving Ortho-K treatment.

## Data Availability

The raw data supporting the conclusions of this article will be made available by the authors, without undue reservation.
