# Supplementary material for: Axial length changes and baseline predictors over five years in children treated with orthokeratology lenses: a retrospective cohort study
Source: Front Bioeng Biotechnol. 2026 Aug 12;14:1870246. doi: 10.3389/fbioe.2026.1870246 (PMC13506842; doi:10.3389/fbioe.2026.1870246)
Supplement: Supplementary file 1 [file DataSheet1.pdf]

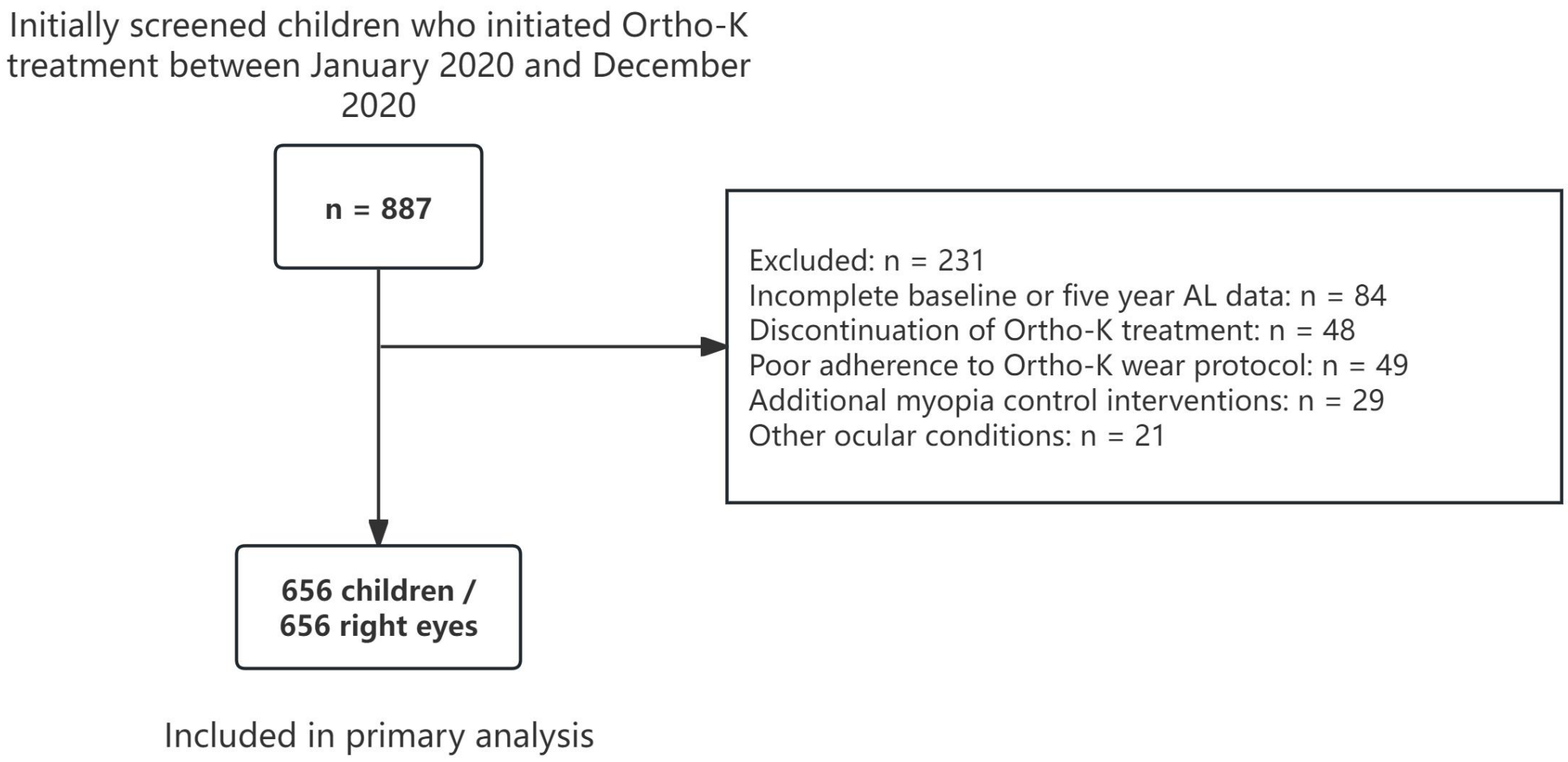

Figure S1. Patient flow diagram of the study cohort. A total of 887 children who initiated orthokeratology treatment between January 2020 and December 2020 were initially screened. Among them, 231 were excluded because of incomplete baseline or five-year axial length data, discontinuation of orthokeratology treatment, poor adherence, additional myopia control interventions, or other ocular conditions. The final analytic cohort included 656 children, with the right eye of each child included in the primary analysis.

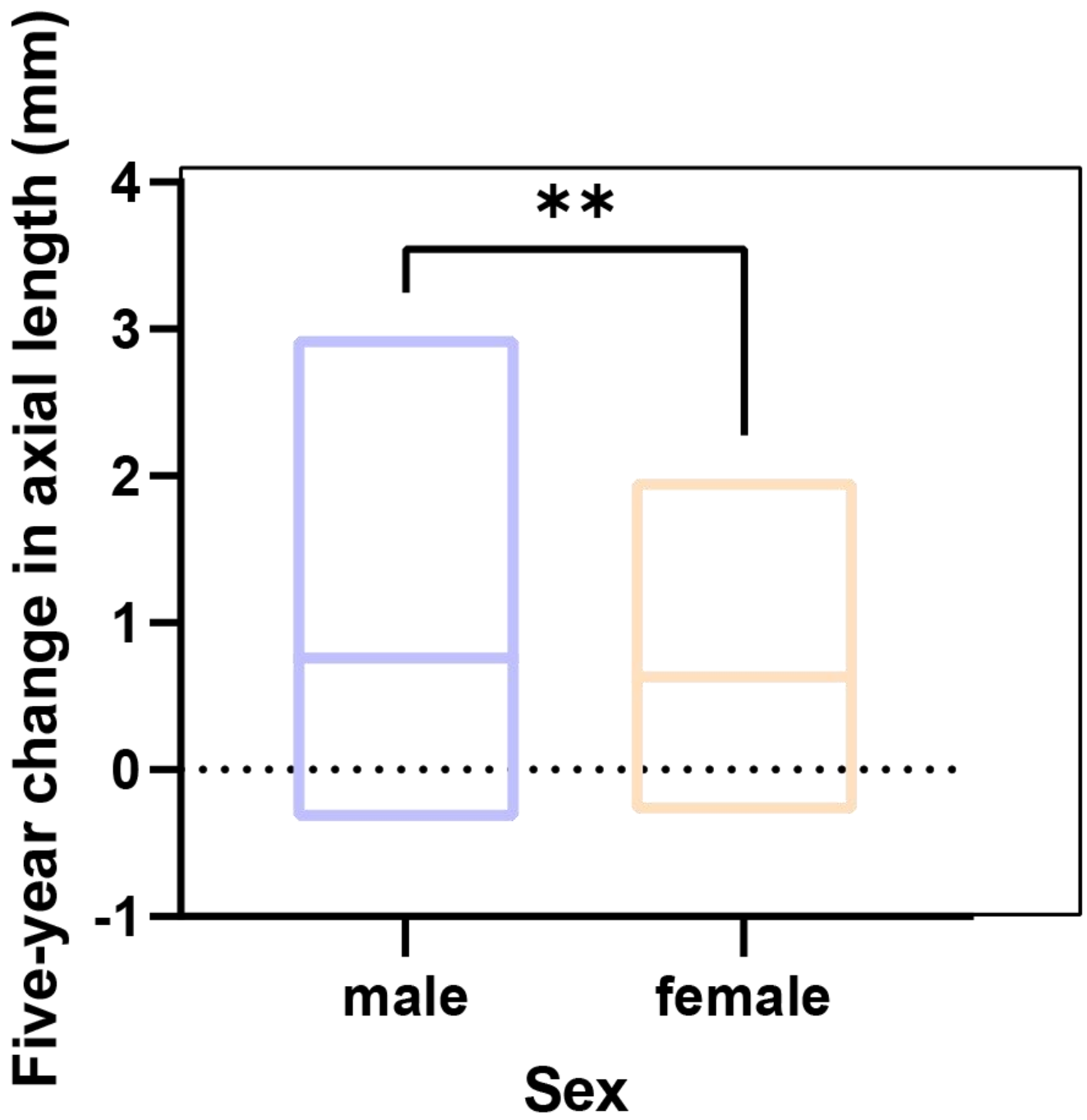

Figure S2. The boxplots of differences in change AL between sex.

We conducted a binary classification of sex, with 294 children classified as male and 362 as female. The middle line of the box plot represents the median values, with the upper and lower lines indicating the maximum and minimum values, respectively. Mann-Whitney test was employed to examine differences in change AL values between sex. The results showed P = 0.0086.

Table S1. Multi-linear Regression Analysis of sex, age, SE, base AL versus AL changes over five years

	VIF	95% CI	β	P
		0.8665 - 3.311	2.089	0.0008
Sex	1.128	-0.1799 - -0.03963	-0.1098	0.0022
Age	1.067	-0.1531 - -0.1102	-0.1317	<0.0001
SE	1.582	0.03157 - 0.08719	0.05938	<0.0001
Base AL	1.813	-0.04325 - 0.06080	0.008778	0.7405

Table S2. Multi-linear Regression Analysis of sex, age, DS, base AL versus AL changes over five years

	VIF	95% CI	β	P
		0.5943 - 3.081	1.838	0.0038
Sex	1.140	-0.1715 - -0.03096	-0.1013	0.0048
Age	1.069	-0.1546 - -0.1118	-0.1332	<0.0001
DS	1.638	0.04055 - 0.1001	0.07034	<0.0001
Base AL	1.895	-0.03298 - 0.07308	0.02005	0.4582
